# Knee Movement Characteristics of Basketball Players in Landing Tasks Before Onset of Patellar Tendinopathy: A Prospective Study

**DOI:** 10.3389/fspor.2022.847945

**Published:** 2022-07-07

**Authors:** Ru Feng, Thomas M. Best, Lin Wang, Weiwei Gao, Hui Liu, Bing Yu

**Affiliations:** ^1^School of Sports and Health, Nanjing Sport Institute, Nanjing, China; ^2^China Institute of Sports and Health, Beijing Sport University, Beijing, China; ^3^UHealth Sports Medicine Institute, University of Miami, Miami, FL, United States; ^4^Department of Sport Medicine and Rehabilitation, Beijing Sport University, Beijing, China; ^5^Division of Physical Therapy, School of Medicine, The University of North Carolina at Chapel Hill, Chapel Hill, NC, United States

**Keywords:** patellar tendinopathy, movement patterns, risk factor, prospective study, injury prevention

## Abstract

**Background:**

Patellar tendinopathy is one of the most common injuries for basketball players. Jumping and landing movement patterns are potential risk factors for patellar tendinopathy.

**Hypothesis:**

Male college basketball players who developed patellar tendinopathy would demonstrate greater peak vertical ground reaction force and knee flexion angular velocity, and smaller knee flexion range of motion and knee flexion angles at initial contact compared to players who did not develop the injury when performing a stop-jump task within a year prior to the onset of the injury.

**Study Design:**

Prospective study.

**Methods:**

Freshmen college basketball male players (*n* = 181) were recruited for three consecutive years and followed to the end of the third year of the study. Three-dimensional kinematic and kinetic data during a stop-jump task were collected for all participants at the beginning of each school year. Peak vertical ground reaction force, knee flexion angle at initial foot contact with the ground, range of motion for knee flexion and maximal knee flexion angular velocity during the landing phases of the stop-jump task were collected and calculated. Development of patellar tendinopathy was monitored in follow-up. Independent t-tests and Cohen's d effect sizes (ES) were used to compare movement patterns between injury and no injury groups for each school year.

**Results:**

A total of 60 knees developed patellar tendinopathy. The injury groups had a significantly greater peak vertical ground reaction force in freshmen and junior years (*P* = 0.020, ES = 0.13; *P* = 0.046, ES = 0.17), smaller knee flexion ROM in freshmen year (*P* = 0.002, ES = 0.10), and greater maximum knee flexion angular velocity in freshmen and junior year (*P* = 0.012, ES = 0.10; *P* = 0.001, ES = 0.35) during the horizontal landing phase before the takeoff of the jump compared to the no injury groups. The injury groups also had a significantly smaller knee flexion angle at initial contact during vertical landing phase after the takeoff of the jump in freshmen and junior years (*P* = 0.001, ES = 0.36; *P* = 0.001; ES = 0.37) during vertical landing phase.

**Conclusion:**

Peak vertical ground reaction force, knee flexion angle at initial foot contact, knee flexion range of motion, and maximum knee flexion angular velocity are associated with patellar tendinopathy among male college basketball players in different school years.

## Introduction

Patellar tendinopathy is one of the most commonly seen injuries in sports in which athletes repeatedly perform explosive jumps, especially for male athletes (David, 1989; Stuart and Peter, 2003; Florit et al., 2019). The reported prevalence of patellar tendinopathy is up to 45% among elite volleyball players and 32% among elite basketball players in cross-sectional studies (Lian et al., 2005; Zwerver et al., 2011). The injury results in substantial time loss and decreased performance forcing up to 53% of athletes to terminate their sports careers (Kettunen et al., 2002). The consequences of this condition suggest an ongoing need for more effective prevention and treatment programs.

To effectively prevent patellar tendinopathy and improve quality of rehabilitation strategies, modifiable injury risk factors need to be identified. Recognizing that patellar tendinopathy is an overuse injury resulting from repetitive stress on the tendon (Magnusson et al., 2010), movement patterns were proposed as risk factors for the injury (Kountouris and Cook, 2007). A previous study found that peak patellar tendon force approaches 7 times body weight during horizontal landing and 5 times body weight during vertical landing in a stop-jump task (Edwards et al., 2012). Studies have shown that individuals with patellar tendinopathy demonstrated significant differences in lower extremity movement patterns in jumping and landing tasks in comparison to healthy individuals (Bisseling et al., 2008; Lin et al., 2009; Couppe et al., 2013; Mann et al., 2013; Scattone Silva et al., 2017). Studies have also revealed that athletes with a history of patellar tendinopathy demonstrated significant differences in lower extremity biomechanics in landing tasks in comparison to those without history of the injury (Bisseling et al., 2007; Sorenson et al., 2010). A recent systematic review suggests that knee and hip joint flexion mechanics in jump-landing are most like risk factors for patellar tendinopathy, and that assessment should include a whole jump-landing task incorporating a horizontal landing, and performed before prospectively to identify risk factors (Harris et al., 2020a). These results indicate that the a stop-jump task that includes horizontal landing, jump, and vertical landing is more appropriate as a test for identifying risk factors for patellar tendinopathy in comparison to the drop jump test that does not include a horizontal landing component.

Only two prospective studies with inconclusive results on biomechanical risk factors of patellar tendinopathy, however, are present in the literature (Visnes et al., 2013; Van der Worp et al., 2016). One study had only 3 of the original 49 participants develop patellar tendinopathy after a follow up of two competitive seasons (Van der Worp et al., 2016). No biomechanical risk factors were identified because of the small number of injuries. The second study only recorded vertical jump height and concluded that great jumping ability was a risk factor for developing patellar tendinopathy, which is not meaningful to the injury prevention (Visnes et al., 2013). High-quality prospective studies with larger sample sizes and more rigorous study designs are essential to definitively determine whether landing biomechanics play a role in the development of patellar tendinopathy (Tayfur et al., 2021).

The primary purpose of this prospective study was to determine knee movement characteristics during the stop-jump task of male basketball players prior to onset of patellar tendinopathy. A previous study reported that most differences in movement patterns between control and pathological patellar tendon populations were in the horizontal landing (Vander Worp et al., 2014). A stop-jump task includes both horizontal and vertical landings similar to rapid acceleration and repetitive landing movements in basketball games and practices (Chappell et al., 2002). and therefore was chosen as the test task in this study. A stop-jump task consists an approach run, a horizontal landing phase, a jump phase, a flight phase and a vertical landing phase (Lin et al., 2005; Edwards et al., 2012). We hypothesized that male college basketball players who developed patellar tendinopathy would have different lower extremity movement patterns within a year prior to the onset of the injury compared to their counterparts who did not develop the injury. A recent systematic review identified 37 variables that may be associated with patellar tendinopathy (Harris et al., 2020a). We selected four biomechanical variables and specifically hypothesized that male college basketball players who developed patellar tendinopathy would have (1) greater peak vertical ground reaction forces, (2) a smaller knee flexion angle at initial foot contact with the ground, (3) a smaller range of motion for knee flexion, and (4) a greater maximal knee flexion angular velocity during the horizontal and vertical landing phase of a stop-jump task within a year prior to the onset of the injury compared to players who did not clinically develop the injury. These four variables were chosen as the dependent variables in this study because (1) they are direct biomechanical measures easy to obtain, (2) they can be directly modified through training, and (3) they are associated with patellar tendon loading (Stanish et al., 1985; Lin et al., 2005, 2009; Bisseling et al., 2007; Chen et al., 2008; Couppe et al., 2013; Van der Worp et al., 2016).

## Methods

### Participants

**Figure 1:**
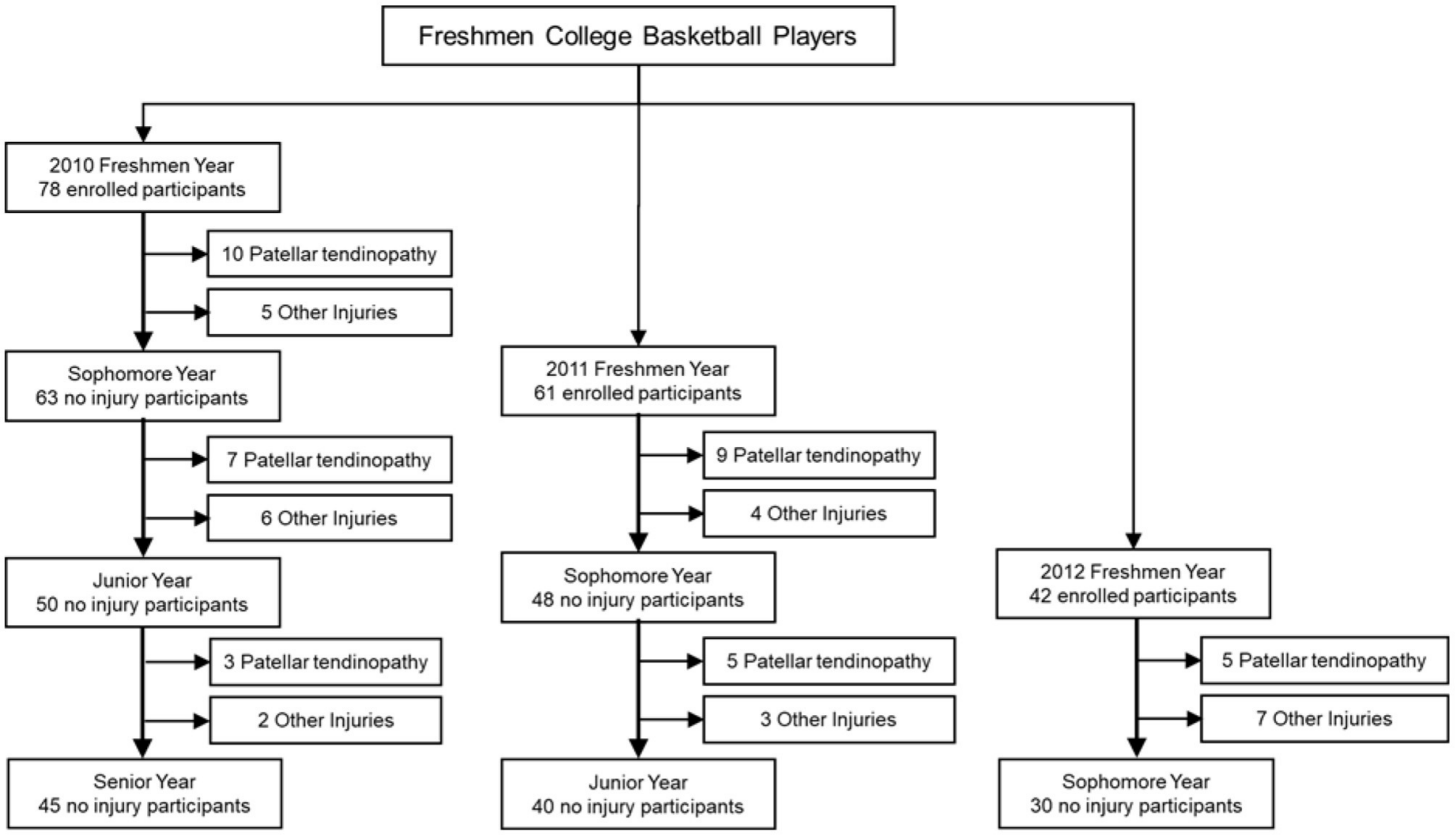
Enrollment of participants.

A total of 256 male college students majoring in sports training of basketball were recruited at the beginning of the fall semester of their freshmen years from 2010 to 2012. Inclusion criteria for enrollment included; (1) absence of patellar tendinopathy at the beginning of the fall semester of their freshmen year, (2) free of any other lower extremity disorders or injuries 6 months prior to the beginning of the fall semester of their freshmen year, (3) free of any lower extremity dysfunction with clinical diagnosis, and (4) no reconstructed lower extremity structures. A total 181 of the 256 recruited participants met the inclusion criteria and were enrolled (Figure 1). Each participant signed a written consent form before any data were collected. The use of human subjects in this study was approved by the Internal Review Board.

### Protocol

At the beginning of each school year, each participant who met the inclusion criteria completed an injury questionnaire and a clinical evaluation of both lower extremities. Patellar tendinopathy was defined as pain within the patellar tendon and a VISA score below 67 (Visentini et al., 1998). All injuries were diagnosed by a sports medicine physician specialized in treatment of lower extremity injuries following recommended clinical procedures (Sorenson et al., 2010). Participants who were free of lower extremity injuries then completed a biomechanical test before the school year's regular training, and subsequently followed through the school year. Enrollment was terminated if a participant reported anterior knee pain during the study and was confirmed to have patellar tendinopathy through clinical evaluation at any time. Participants who were diagnosed with other injuries that prevented them from completing their regular training were also excluded from the study.

For biomechanical testing at the beginning of each school year each participant was asked to wear spandex shorts, a T-shirt, and basketball shoes to perform the stop-jump task. A video clip was played to demonstrate the stop-jump task to each participant. The task included an approach run of about 5 m followed by a two-footed landing with each foot on separate force plates, and an immediate two-footed takeoff for maximum height followed a second two-footed landing with each foot on a force plate. After a 10 min warm up including jogging and stretching, reflective markers were attached bilaterally to the anterior superior iliac spine (ASIS), anterior aspect of the thigh, medial and lateral femur condyles, tibial tuberosity, medial and lateral malleolus, toe, and heel. An additional marker was also attached at the L4-5 vertebral body level. After a standing calibration trial, markers on the medial femoral condyles and medial malleolus were removed. Participants was then instructed to perform three trials of the stop-jump task with their full effort for maximum jumping height. A successful trial was defined as one in which the participant performed the stop-jump task as required and all videographic and force plate data were collected.

### Data Collection

The three-dimensional coordinates of reflective markers were collected at a sample rate of 200 frames/second using a videographic data collection system with eight video cameras (Motion Analysis Corporation, USA). Ground reaction force signals were recorded at a sample rate of 1,000 samples/channel/second using two force plates (Kistler Instrument AG, Switzerland). Videographic and force plate data collection were time synchronized using a Cortex acquisition system (Motion Analysis Corporation, Santa Rosa, CA, USA).

### Data Reduction

The raw three-dimensional coordinates of reflective markers were filtered through a Butterworth lower-pass digital filter at a cut-off frequency of 13 Hz (Yu et al., 1999). The coordinates of the hip joint center were calculated from the coordinates of the markers on the ASISs and L4–L5 joint (Bell et al., 1990). Joint angles were reduced as Cardan angles of the distal segment reference frames relative to the proximal segment reference frames as recommended by International Society of Biomechanics (Cole et al., 1993). Knee joint angular velocities were also calculated as the time-derivatives of joint angles (Haug, 1992). Three-dimensional ground reaction forces were calculated from ground reaction force signals. Peak vertical ground reaction force was identified for each landing phase. Peak vertical ground reaction forces were normalized to body weight (BW).

**Figure 2:**
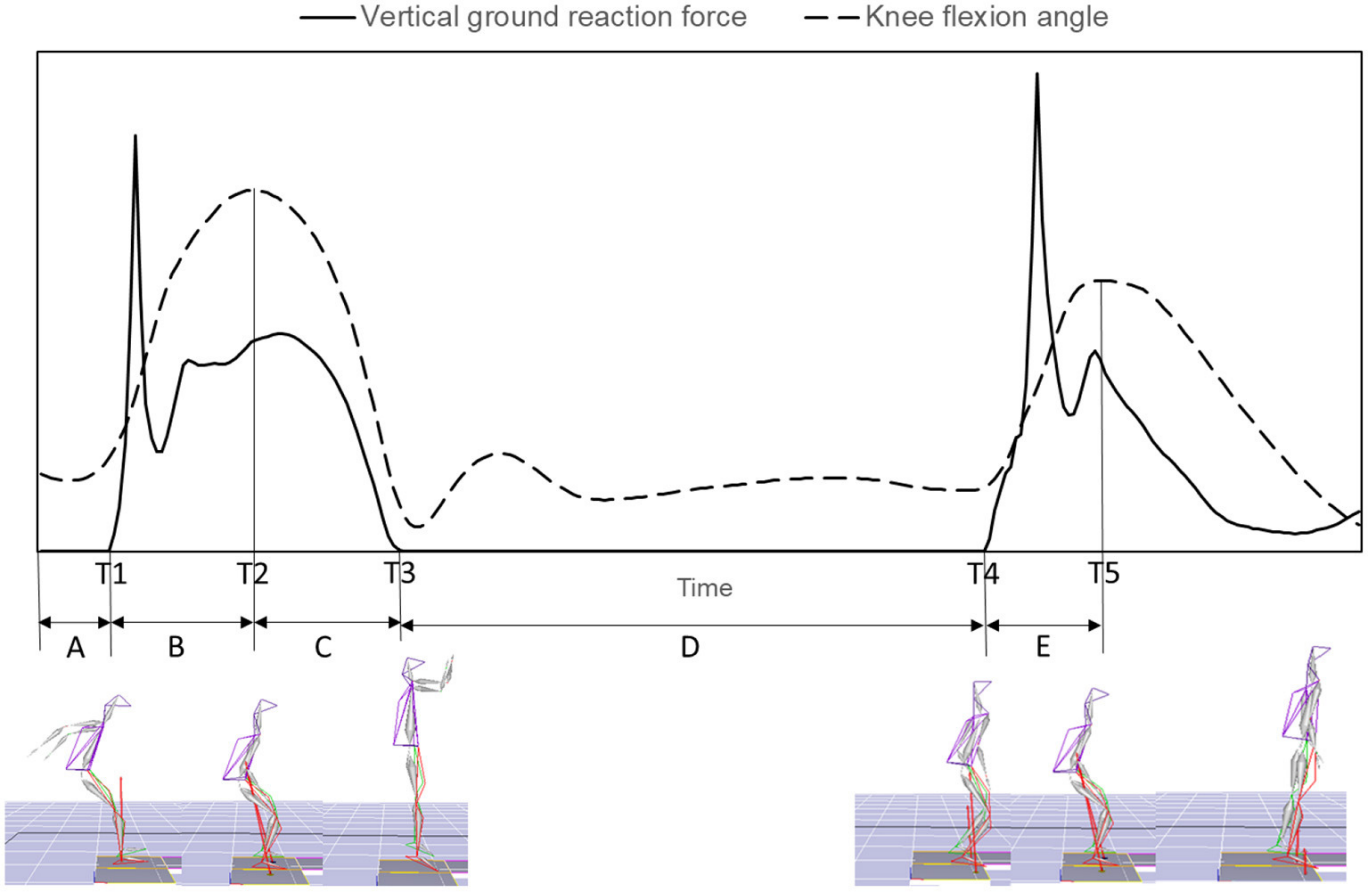
Definitions of instants and phases: T1, initial foot contact of horizontal landing; T2, maximum knee flexion of horizontal landing; T3, takeoff of vertical jump; T4, initial foot contact of vertical landing; T5, maximum knee flexion of vertical landing; **(A)** approach run phase; **(B)** horizontal landing phase; **(C)** jump phase; **(D)** flight phase; **(E)** vertical landing phase.

Specific instants of initial foot contact of horizontal landing, maximum knee flexion of horizontal landing, takeoff of vertical jump, initial foot contact of vertical landing, and maximum knee flexion of vertical landing were identified (Figure 2). The horizontal landing, jump, flight, and vertical landing phases were defined (Figure 2). An initial foot contact was identified as the time represented by the first frame in which the vertical ground reaction force was greater than zero immediately after last step of the approach run or flight. The takeoff of the vertical jump was identified as the time represented by the first frame in which the vertical ground reaction force was zero immediately after the initial foot contact with the ground of the horizontal landing phase. Range of motion (ROM) for knee flexion was calculated as the difference between the maximum and minimum of the flexion-extension angle of knee joint during each landing phase.

### Data Analysis

Participants developed lower extremity injuries other than patellar tendinopathy were excluded in analysis. All legs of the participants included in data analysis were assigned to either the no injury group or the patellar tendinopathy group. The no injury group in each school year included all legs that had not developed patellar tendinopathy before the completion of biomechanical testing each school year. The patellar tendinopathy groups in each school year included all legs that developed the injury after biomechanical testing at the beginning of each school year and before completion of testing of the following school year. Lower extremity kinematics and kinetics of each leg collected at the beginning of the fall semester of each given school year were used for analyses to represent the biomechanical characteristics of the participants in the given school year group.

To test our hypotheses, independent t-tests were performed to compare dependent variables between groups in each school year. A Type I error rate no >0.05 was chosen as the indication of statistical significance. Cohen's d effect sizes (ES) were calculated to determine the magnitude of the differences for all comparisons. A difference was considered as large if ES ≥ 0.5, as medium if 0.5 > ES ≥ 0.2, and as small if ES <0.2. All statistical analyses were performed using version 20.0 of the SPSS computer program package for statistical analysis (IBM, Armonk, NY, USA).

## Results

A total of 181 participants were enrolled in the study in their freshmen year. 16 players in freshmen year, 9 players in sophomore year, and 2 players in junior year had non patellar tendinopathy knee disorders or injuries during follow up (Table 1).

**Table 1:** Non-patellar tendinopathy disorders or injuries in 3 years of follow-up.

**Injury type**	**Number**
Chondromalacia patellae	8
Meniscus injury	3
Injury of knee collateral ligament	5
Injury of anterior cruciate ligament	2
Ankle joint injury	9
Total	27

A total of 165 participants in their freshmen year, 102 participants in their sophomore year, and 48 participants as juniors were included in the data analysis (Table 2). 40 of 330 legs developed patellar tendinopathy in freshmen year, 15 of 204 legs developed the injury in sophomore year, and 5 of 96 legs developed patellar tendinopathy in junior year (Table 2).

**Table 2:** Characteristics of participants included in data analysis in each school year.

**School year**	**Number of participants**	**Body mass (kg)**	**Standing height (m)**	**Age (years)**	**Length of training (years)**	**Not injured (legs)**	**Patellar tendinopathy**	
							**Injured (legs)**	**Injury rate (%)**
Freshmen	165	78.9 ± 11.3	1.86 ± 0.06	19.3 ± 0.9	5.5 ± 2.7	290	40	12.1
Sophomore	102	80.4 ± 11.1	1.86 ± 0.06	20.3 ± 1.1	6.1 ± 2.7	189	15	7.4
Junior	48	80.9 ± 11.1	1.87 ± 0.06	21.1 ± 1.1	7.2 ± 3.0	91	5	5.2

### Comparisons of Movement Patterns in Horizontal Landing Phase

Independent t-tests revealed that the patellar tendinopathy group had significantly greater peak vertical ground reaction force compared to no injury group in freshmen year (*P* = 0.020, ES = 0.13) and junior year (*P* = 0.046, ES = 0.17).

Independent t-tests also revealed that the patellar tendinopathy group had smaller knee joint flexion range of motion in freshmen year (*P* = 0.002, ES = 0.10).

Independent t-test further revealed that the patellar tendinopathy group had greater maximum knee flexion angular velocity compared to no injury group in freshmen year (*P* = 0.012, ES = 0.10) and junior year (*P* = 0.001, ES = 0.35) (Table 3).

**Table 3:** Comparisons between no injury and patellar tendinopathy groups in male players (*P*-value ≤ 0.05 was considered as statistical significance. A difference was considered as large if Cohen's *d* value ≥ 0.05, as medium if 0.05 < Cohen's *d* value ≤ 0.02, and as small if Cohen's *d* value <0.02).

**Variable**	**School year**	**Horizontal landing phase**				**Vertical landing phase**			
		**No injury**	**Patellar tendinopathy**	***P*-value**	**Cohen's d value**	**No injury**	**Patellar tendinopathy**	***P*-value**	**Cohen's d value**
Peak vertical ground reaction force (BW)	Freshmen	2.10 ± 0.69	2.38 ± 0.86	**0.020**	**0.13**	2.87 ± 1.00	2.99 ± 0.58	0.451	0.04
	Sophomore	2.17 ± 0.53	2.27 ± 0.48	0.463	0.05	2.75 ± 0.82	2.58 ± 0.60	0.427	0.06
	Junior	2.15 ± 0.56	2.52 ± 0.42	**0.046**	**0.17**	2.72 ± 0.57	2.87 ± 0.35	0.538	0.06
Knee flexion angle at initial contact (degree)	Freshmen	26.0 ± 7.5	28.5 ± 10.1	0.066	0.09	19.2 ± 8.2	19.5 ± 8.6	0.810	0.02
	Sophomore	32.0 ± 10.1	33.3 ± 10.2	0.615	0.04	24.7 ± 7.5	16.0 ± 11.6	**0.001**	**0.36**
	Junior	32.1 ± 10.2	35.1 ± 0.93	0.521	0.09	25.6 ± 6.1	16.3 ± 6.6	**0.001**	**0.37**
Knee flexion range of motion (degree)	Freshmen	64.5 ± 12.2	58.4 ± 7.0	**0.002**	**0.10**	53.5 ± 11.4	56.2 ± 14.0	0.530	0.02
	Sophomore	56.3 ± 11.5	60.2 ± 12.0	0.203	0.07	54.5 ± 11.3	53.7 ± 11.9	0.282	0.06
	Junior	54.6 ± 10.9	57.1 ± 13.6	0.625	0.05	53.8 ± 11.9	45.9 ± 6.8	0.537	0.06
Maximum knee flexion angular velocity (degree/s)	Freshmen	565.9 ± 78.1	625.0 ± 79.7	**0.012**	0.10	543.4 ± 113.6	556.1 ± 125.1	0.844	0.01
	Sophomore	614.0 ± 60.2	664.3 ± 34.2	0.148	0.08	543.3 ± 117.3	540.8 ± 120.9	0.508	0.06
	Junior	571.8 ± 93.7	756.9 ± 60.4	**0.001**	**0.35**	577.3 ± 123.3	541.0 ± 211.0	0.214	0.19

*Values are mean ± SD. The bold values represent significant difference between no injury group and patellar tendinopathy group*.

### Comparisons of Movement Patterns in Vertical Landing Phase

Independent t-tests finally revealed that the patellar tendinopathy group had significantly smaller knee flexion angle at initial foot contact during vertical landing compared to the no injury group in sophomore year (*P* = 0.001, ES = 0.36) and junior years (*P* = 0.001, ES = 0.37) (Table 3).

## Discussion

This study determined lower extremity biomechanical characteristics of male college basketball players who developed patellar tendinopathy within a year of variable measurement. Our results support our hypothesis that male college basketball players who developed patellar tendinopathy would have different lower extremity movement patterns within a year prior to the onset of the injury compared to those who did not develop the injury. Players who developed the injury in freshmen and junior years had a greater peak vertical ground reaction force during vertical landing phase of the stop-jump task compared to their counterparts who did not sustain the injury. Peak vertical ground reaction forces were over 2 times body weight during horizontal landing and nearly 3 times body weight during vertical landing which were consistent previous studies (Edwards et al., 2012, 2014). Although the peak vertical ground reaction forces during the horizontal landing phase were lower compared to the vertical landing phase in the stop-jump task, the patellar tendon forces might be greater in the horizontal landing phase compared to the vertical landing phase (Edwards et al., 2012). A recent study showed that volleyball players with a history of patellar tendinopathy demonstrate lower peak vertical ground reaction force during horizontal landing in a stop-jump task compared to those without histories of patellar tendinopathy, interpreted as an adaptation to avoid pain due to injury (Sorenson et al., 2010). Increasing vertical ground reaction force in the horizontal landing may increase the knee extension moment and thus patellar tendon loading due to the correlations among vertical and horizontal ground reaction forces and knee extension moment during landing movements (Lin et al., 2005, 2009). Our results support the findings of a previous study showing peak vertical ground reaction force is a predictor for developing patellar tendinopathy (Fietzer et al., 2012).

The results also showed that male college basketball players who developed patellar tendinopathy in their freshmen year had significantly smaller knee flexion ROM during the horizontal landing phase within a year prior to the onset of the injury compared to their counterparts who did not develop the injury. Small knee flexion ROM is a characteristic of stiff landing associated with increased knee extension moment (Chen et al., 2008; Van der Worp et al., 2016;). As previous discussed, increasing knee extension moment increases patellar tendon loading. Increased strain of the collagen fibers within the patellar tendon may lead to tensile failure and contribute to patellar tendinopathy (Couppe et al., 2013).

We also showed that participants who developed patellar tendinopathy in their freshmen and junior years had greater maximum knee flexion angular velocity during the horizontal landing phase within a year prior to the onset of the injury compared to their counterpart who did not develop the injury. Risk for patellar tendinopathy is thought to be greatest during the eccentric phase of landing (Vander Worp et al., 2014). A previous study found that volleyball players with a history of patellar tendinopathy but no symptoms had a significantly greater knee flexion velocity compared to those who had no history of patellar tendinopathy during vertical landing (Bisseling et al., 2008). The patellar tendon is elongated and loaded while the knee is flexing during landing. Eccentric loading can results in force magnitudes three times those observed during concentric loading (Stanish et al., 1985). Higher elongation speed typically results in greater tendon strain (Earp et al., 2016). Repeated high speed eccentric loading is believed to be a primary cause of cumulative tendon micro trauma (Stanish et al., 1985). Fast repetitive stretch on the tendon especially during the horizontal landing phase is considered an important risk factor for patellar tendinopathy (Richards et al., 1996; Vander Worp et al., 2014; Earp et al., 2016).

Subjects who developed patellar tendinopathy in their sophomore and junior years had smaller knee flexion angle at initial foot contact with the ground during the vertical landing phase compared to their counterparts who did not develop the injury. Landing with a small knee flexion angle may also result in an increase in quadriceps force (Earp et al., 2016; Harris et al., 2020a), and thus an increase in patellar tendon strain that may lead to tensile failure and contribute to patellar tendinopathy (Bisseling et al., 2007).

Testing movement characteristics every year is a unique strength of the present study. Our results showed that participants in different school years apparently have different movement characteristics associated with patellar tendinopathy. This finding is consistent with literature showing movement patterns may change over time with age and training (Padua et al., 2011; Campa et al., 2019). Ideally, movement testing would be performed as close to the onset of the injury as possible to establish a clear association between the risk factor and injury. We collected movement data for all participants at the beginning of each school year in the present study and school year was included as an independent variable to minimize the effects of training on movement patterns and outcome. The findings of this study set the basis for further studies on risk factors predicting development of patellar tendinopathy.

Lack of imaging confirmation of clinically diagnosed patellar tendinopathy may be a limitation of this study. Patellar tendinopathy cases in this study were diagnosed by an experienced sports medicine physician hopefully minimizing the risk of overdiagnosis of the problem. A recent study, however, showed that that the clinical diagnosis of patellar tendinopathy may lack the sensitivity to differentiate patellar tendinopathy and patellofemoral pain (Harris et al., 2020b). Clinically diagnosed patellar tendinopathy, therefore, may need to be confirmed with the use of imaging in future studies (Lian et al., 2003).

## Conclusions

Male college basketball players who showed greater peak ground reaction force and maximum knee flexion velocity together with smaller range of knee flexion in horizontal landing have an increased risk of developing patellar tendinopathy. Players who showed smaller knee flexion angle at initial contact with the ground in vertical landing may also have an increased risk of developing patellar tendinopathy. Risk factors for patellar tendinopathy may be different for male college basketball players in different school years.

## Data Availability Statement

The raw data supporting the conclusions of this article will be made available by the authors, without undue reservation.

## Ethics Statement

The studies involving human participants were reviewed and approved by Scientific Research Internal Review Board of Beijing Sport University. The patients/participants provided their written informed consent to participate in this study.

## Author Contributions

Material preparation, data collection, and analysis were performed by RF, TB, LW, WG, HL, and BY. The first draft of the manuscript was written by RF. All authors contributed to the study conception, design, and commented on previous versions of the manuscript. All authors read and approved the final manuscript.

## Funding

This research was partially supported by China National Natural Science Foundation (Grant No. 12132009).

## Conflict of Interest

The authors declare that the research was conducted in the absence of any commercial or financial relationships that could be construed as a potential conflict of interest.

## Publisher's Note

All claims expressed in this article are solely those of the authors and do not necessarily represent those of their affiliated organizations, or those of the publisher, the editors and the reviewers. Any product that may be evaluated in this article, or claim that may be made by its manufacturer, is not guaranteed or endorsed by the publisher.
